# Association between serum progesterone levels on the day of frozen-thawed embryo transfer and pregnancy outcomes after artificial endometrial preparation

**DOI:** 10.1186/s12884-023-05596-4

**Published:** 2023-05-30

**Authors:** Qianqian Zhu, Jialyu Huang, Yue Lin, Liyao Jiang, Xuefeng Huang, Jing Zhu

**Affiliations:** 1 Department of Assisted Reproduction, Shanghai Ninth People’s Hospital, Shanghai Jiaotong University School of Medicine, Shanghai, China; 2 Center for Reproductive Medicine, Jiangxi Maternal and Child Health Hospital, Nanchang University School of Medicine, Nanchang, China; 3 Reproductive Medicine Center, The First Affiliated Hospital of Wenzhou Medical University, Wenzhou, China; 4 Department of Obstetrics and Gynecology, Wenzhou Lucheng People’s Hospital, Wenzhou, China

**Keywords:** Progesterone, Dydrogesterone, Live birth rate, Frozen embryo transfer, Artificial cycle

## Abstract

**Background:**

Previous studies have examined that a range of optimal serum P level during the implantation period was associated with optimal live birth rates. However, those results obtained with vaginal or intramuscular route of progesterone administration for luteal phase support (LPS) alone. Is there a relationship between the serum progesterone (P) on the day of frozen-thawed embryo transfer (FET) with the likelihood of a live birth (LB) in artificial cycles (AC) when using a combination of oral dydrogesterone and vaginal progesterone for LPS?

**Methods:**

This was a retrospective study of 3659 FET cycles with artificial endometrial preparation in a Chinese tertiary-care academic medical centre from January 2015 to February 2017. Endometrial preparation was performed using estradiol (E_2_) valerate (Fematon-red tablets) 8 mg/d beginning on day 3 of the cycle, followed by administration of P both orally (8 mg/d Fematon-yellow tablets) and vaginally (400 mg/d; Utrogestan). The primary endpoint was live birth rate (LBR). The association between the serum P level on the embryo transfer day and pregnancy outcomes was evaluated by univariable and multivariable logistic regression analysis.

**Results:**

The LBRs according to the serum P quartiles were as follows: Q1: 35.7%; Q2: 37.4%; Q3: 39.1% and Q4: 38.9%. Logistic regression analysis showed that the odds of a LB were not significantly different between the low (P < 7.9 ng/mL) and high (P ≥ 7.9 ng/mL) progesterone groups before or after adjustment (crude OR = 0.89, 95% CI: 0.76–1.04; adjusted OR = 0.89, 95% CI: 0.75–1.04).

**Conclusion:**

The present study suggests that the serum P levels on the day of embryo transfer (ET) do not correlate with the likelihood of a LB in artificial cycles when using a combination of oral dydrogesterone and vaginal progesterone for luteal phase support.

**Supplementary Information:**

The online version contains supplementary material available at 10.1186/s12884-023-05596-4.

## Background

Since the first live birth by frozen-thawed embryo transfer (FET) in 1983, embryonic cryopreservation and FET procedures have progressively advanced and become essential tools in the treatment of infertility [1, 2]. Elective embryo cryopreservation was mainly developed to prevent ovarian hyperstimulation syndrome in women who were at risk [2–4], but its use has been extended to cycles with preimplantation diagnoses and embryo-endometrial asynchrony [5, 6]. FET is performed using different cycle regimens: spontaneous ovulatory (natural) cycles; cycles in which the endometrium is artificially prepared with oestrogen and progesterone hormones, commonly known as hormonally substituted artificial cycles (AC); and cycles in which ovulation is induced by drugs (ovulation induction FET cycles) [7]. However, the most effective method to prepare the endometrium prior to FET is still a matter of debate. Hormonally substituted AC-FET is commonly used, requires less monitoring than other FET protocols and thus offers flexibility in terms of the timing of the thaw and transfer of the embryo [8].

The importance of progesterone in the establishment and maintenance of pregnancy has been proven by interventional studies in early pregnancy, which showed that progesterone deficiency caused by a lutectomy or by blocking the actions of progesterone (using a progesterone antagonist) lead to pregnancy loss [9, 10]. In AC-FET, administration of oestrogen and progesterone is necessary due to the lack of a corpus luteum and endogenous sex steroid production [8].

Multiple routes of P administration are available. Over the last few decades, vaginal and intramuscular routes have been preferentially used, whereas oral P has been generally avoided due to poor bioavailability and inferior assisted reproductive technology (ART) outcomes [11–13]. Surveys have indicated that when given the choice, most patients prefer vaginal over intramuscular P administration for a variety of reasons, including greater convenience, ease of use, and less pain [14, 15]. Dydrogesterone is a synthetic progestin with enhanced oral bioavailability that is highly selective for the progesterone receptor [16, 17]. Furthermore, data from prospective trials for luteal phase support in IVF show that oral dydrogesterone is as effective as micronized vaginal progesterone (MVP), is well tolerated overall, and has a higher patient satisfaction rate than MVP [16, 18].

Recently, it has been reported that a range of serum progesterone concentrations during the implantation period is associated with an optimal live birth rate [19–25]. Although these results are controversial, they suggest an optimal serum P threshold for the LBR of artificial cycles when using intravaginal or intramuscular progesterone; however, different cut-off levels have been suggested. Our in vitro fertilization (IVF) centre applies a combination of oral dydrogesterone and vaginal progesterone for LPS in AC-FET. We are interested in determining whether the findings are consistent under the combination of oral dydrogesterone and vaginal progesterone administration. Given this background, the present study aimed to examine whether serum P levels on the day of ET are related to the live birth rate for artificial cycles when using a combination of oral dydrogesterone and vaginal progesterone for LPS.

## Methods

### Study design and patients

This retrospective cohort study was conducted at the Department of Assisted Reproduction of the Ninth People’s Hospital of Shanghai Jiao Tong University School of Medicine (Shanghai, China) from January 2017 to February 2019. It was approved by the Ethics Committee (Institutional Review Board) of the Ninth People’s Hospital of Shanghai. All participants provided informed consent after counselling for infertility treatments and routine IVF procedures. All patients underwent artificial endometrial preparation with a combination of oral dydrogesterone and vaginal progesterone for LPS only. Inclusion criteria were as follows: 19 < age < 50 years; BMI < 30 kg/m2; and no systemic diseases. Due to the potential bias from recurrent implantation failure (the failure of clinical pregnancy after 4 good quality embryo transfers, with at least three fresh or frozen IVF cycles in women under the age of 40) [26], analysis was limited only to patients undergoing their first or second FET cycles. The exclusion criteria were recurrent miscarriages (the loss of three or more consecutive pregnancies (< 28 weeks of gestation), infertility due to severe male factors (e.g., oligozoospermia, cryptozoospermia and azoospermia), uterine diseases (e.g., fibroids, polyps, and previously diagnosed Müllerian abnormalities) or the presence of hydrosalpinx, women whose triple-line endometrium of < 7 mm thickness after as many days as 21 E_2_ administration. Likewise, records with missing data were excluded. Therefore, a final cohort of 3659 cycles was analysed.

### Protocol of endometrial preparation and embryo transfer

Details on endometrial preparation procedures have been described in our previous study [27]. Endometrial preparation was induced with sequential provision of oral E_2_ (Fematon-red tablets, Abbott Biological, USA) 8 mg/d from cycle day 3 onwards. After 12–14 days of oestrogen therapy, a blood sample was collected, and a vaginal ultrasound were performed for measurements of oestradiol, LH, and progesterone levels and endometrial thickness, respectively. If the endometrial thickness was < 7 mm, oestrogen therapy was extended for as long as 7 days if required. If a triple-line endometrium of ≥ 7 mm thickness was observed with serum progesterone concentrations < 1.0 ng/ml, P was administered both orally (40 mg dydrogesterone and 8 mg E twice per day; Fematon-yellow tablets, Abbott Biologicals, USA) and vaginally (400 mg/d; Utrogestan, Besins Manufacturing, Belgium). If a triple-line endometrium of ≥ 7 mm thickness was observed with serum progesterone concentrations ≥ 1.0 ng/ml, the FET cycle should be cancelled due to premature ovulation. The time of thaw and transfer was set as the 3rd or 5th day after P administration depending on the embryo stage. The maximum number of embryos transferred was two per patient in each FET cycle. Given the absence of corpora lutea, exogenous P4 administration was continued until 10-week gestation. A blood sample was obtained between 7 and 8 am on the day of frozen-thawed embryo transfer for serum oestradiol and progesterone measurements. Embryos were generated from either IVF or intracytoplasmic sperm injection cycles and were vitrified and warmed as previously described [28, 29] on day 3 or at the blastocyst stage. In brief, the Cummins criteria and the Gardner and Schoolcraft grade system were used to grade D3 embryos and blastocysts respectively [30, 31]. We defined top-quality embryo as grade I 7–10 cells on Day 3 and grade 4BB or higher on Day 5/6. All embryo transfers were performed under ultrasound guidance. The number of transferred embryos and the stage of the embryo were recorded. A human chorionic gonadotrophin (HCG) test was measured 12 or 14 days after embryo transfer and ultrasound examination was routinely performed 28 days after embryo transfer.

### Serum hormonal assays

Hormone levels were measured by chemiluminescence (Abbott Biologicals B.V.). Intra- and inter-assay coefficients of variation were 7.9 and 10% for P. The synthetic progestogens used (dydrogesterone) did not have any cross-reaction with the progesterone assay.

### Outcome measures

The primary endpoint was the relationship between progesterone levels on the embryo transfer day and the live birth rate per cycle. Secondary endpoints included the implantation rate, positive β-HCG test rate, clinical pregnancy rate, ongoing pregnancy rate at 12 weeks of gestation and early miscarriage (first-trimester pregnancy loss) rate. A human chorionic gonadotrophin (hCG) test was considered positive if HCG was > 10 IU/l. The clinical pregnancy rate (CPR) was defined as the proportion of patients diagnosed by ultrasonographic visualization of one or more gestational sacs or definitive clinical signs of pregnancyafter ET among all transfer cycles. The implantation rate was defined as the number of gestational sacs divided by the number of embryos transferred. The early miscarriage rate was defined as the proportion of patients with spontaneous pregnancy termination before the gestational age of 12 weeks. The ongoing pregnancy rate (OPR) was defined as the proportion of patients with a gestational sac with foetal heart activity assessed by ultrasound examination at 12 weeks of gestation among all transfer cycles.

The live birth rate (LBR) was defined as the proportion of the number of deliveries that resulted in at least one live birth among all transfer cycles. The main neonatal outcomes of the study included preterm birth (PTB), low birthweight (LBW), and major congenital malformations. PTB was defined as a birth takes place after 22 weeks and before 37 completed weeks of gestation. LBW was defined as a birthweight below 2500 g [32, 33]. Major congenital malformations were defined and coded according to the Q codes (Q00–Q99) of the International Classification of Diseases, 10th Revision (ICD-10) [34].

### Statistical analysis

Serum P on the day of ET was classified into four quartiles according to the 25th, 50th and 75th percentiles. Continuous variables were presented as the mean plus/minus standard deviation, and the Kruskal-Wallis test was used to compare continuous variables. Categorical variables were described as the frequency with the rate, and between-group differences were assessed by chi-square test or Fisher’s exact test, as appropriate. The association between serum P on the day of ET and pregnancy outcome was evaluated by univariable and multivariable logistic regression analysis. All potential confounders were introduced into the regression equation for adjustment by the entry method, whether significant differences between groups were observed. These included maternal age (continuous), maternal BMI (continuous), duration of infertility (continuous), type of infertility (primary, secondary), infertility diagnosis (tubal factor, male factor, ovulatory, endometriosis, unexplained or combined), ovarian stimulation protocol (GnRH-a short, mild stimulation or progestin-primed ovarian stimulation (PPOS)), fertilization method (IVF, ICSI or IVF + ICSI), duration of estrogen use (continuous), endometrial thickness (continuous), progesterone level on embryo transfer day, number of embryos transferred (single or double), embryo stage at transfer (cleavage or blastocyst), transfer of ≥ 1 top-quality embryos and duration of embryo cryopreservation (continuous).

All P values were based on two-sided tests, and P < 0.05 was considered statistically significant. Statistical analysis was performed with the Statistical Package for the Social Sciences (version 20.0; SPSS Inc., USA).

## Results

**Fig. 1:**
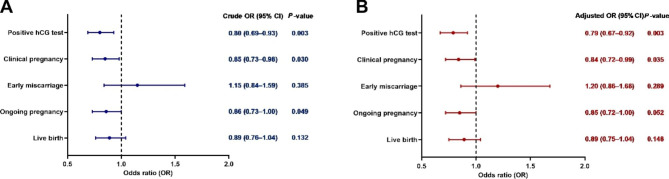
Crude and adjusted odds ratios of pregnancy outcomes in serum progesterone Q1 (< 7.9 ng/mL) group compared with the Q2–Q4 (≥ 7.9 ng/mL) group. Abbreviations: Abbreviations: hCG = human chorionic gonadotropin

A total of 3659 cycles were analysed in terms of demographic characteristics and reproductive outcomes. Serum P on the day of ET was 10.30 ± 3.88 ng/ml. Supplementary Fig. 1 shows the 5th, 10th, 25th, 50th, 75th, 90th and 95th percentile values. The serum P intervals for each quartile were as follows: Q1, < 7.9 ng/ml; Q2, 7.9–9.6 ng/ml; Q3, 9.7–12 ng/ml; and Q4, ≥ 12.1 ng/ml. The baseline characteristics according to the serum P level on the day of ET are shown in Table 1. The four groups differed significantly in terms of the proportions for ovarian stimulation protocol (P < 0.001), endometrial thickness (P = 0.002) and embryo stage at transfer (P = 0.01). No significant differences were found when maternal age, BMI, duration of infertility, infertility diagnosis, fertilization method, number of embryos transferred or duration of embryo cryopreservation were analysed. The mean serum P values on the day of ET were 6.3 ± 1.4, 8.8 ± 0.5, 10.8 ± 0.7 and 15.3 ± 3.8 ng/ml in the Q1, Q2, Q3 and Q4 groups, respectively (P < 0.001).The patients with serum P levels on the day of ET that were < 7.9 ng/ml (Q1) had a significantly lower positive β-HCG test rate before or after adjustment relative to the high progesterone group (Q2-Q4) ((429/905,47.4%) versus (1461/2754,53.0%) (P = 0.003); adjusted OR = 0.79, 95% CI: 0.67–0.92, P = 0.003). The low progesterone group (Q1) also had a significantly lower CPR and OPR than the other patients (Q2–Q4): (393/905,43.4%) versus (1310/2754,47.6%) (P = 0.03); (333/905,36.8%) versus (1115/2754,40.5%) (P = 0.049). However, the odds of OPR between the two groups did not show a significant difference after adjustment (adjusted OR = 0.85, 95% CI: 0.72-1.00). In addition, for women in the low progesterone group (Q1), the odds of a LB did not show a significant difference before or after adjustment relative to the high progesterone group (Q2-Q4) (crude odds ratio [OR] = 0.89, 95% confidence interval [CI]: 0.76–1.04; adjusted OR = 0.89, 95% CI: 0.75–1.04). Furthermore, there was no statistically significant difference in the adjusted odds of early miscarriage between the low and high progesterone groups (Table 2; Fig. 1).

**Table 1:** Baseline characteristics according to serum progesterone levels on the day of embryo transfer

	Progesterone quartiles (ng/mL)				*P*-value
	Q1 (< 7.9)	Q2 (7.9–9.6)	Q3 (9.7–12.0)	Q4 (≥ 12.1)	
No. of cycles	905	894	938	922	
Maternal age (years)	35.1 ± 5.3	35.1 ± 5.2	34.8 ± 5.3	35.5 ± 5.3	0.052
Maternal BMI (kg/m^2^)	21.92 ± 2.73	22.07 ± 2.98	21.75 ± 2.78	21.70 ± 2.87	0.057
Duration of infertility (years)	3.7 ± 2.8	3.9 ± 3.0	3.6 ± 2.9	3.6 ± 2.7	0.067
Primary infertility, *n* (%)	469 (51.8)	438 (49.0)	449 (47.9)	425 (46.1)	0.097
Infertility diagnosis, *n* (%)					0.192
Tubal	438 (48.4)	418 (46.8)	446 (47.5)	426 (46.2)	
Male	94 (10.4)	86 (9.6)	85 (9.1)	80 (8.7)	
Ovulatory	150 (16.6)	138 (15.4)	170 (18.1)	159 (17.2)	
Endometriosis	68 (7.5)	69 (7.7)	70 (7.5)	50 (5.4)	
Unexplained	96 (10.6)	112 (12.5)	108 (11.5)	119 (12.9)	
Combined	59 (6.5)	71 (7.9)	59 (6.3)	88 (9.5)	
Ovarian stimulation protocol, *n* (%)					< 0.001
Mild stimulation	187 (20.7)	134 (15.0)	152 (16.2)	121 (13.1)	
GnRH-agonist short	50 (5.5)	49 (5.5)	30 (3.2)	39 (4.2)	
Progestin-primed ovarian stimulation	668 (73.8)	711 (79.5)	756 (80.6)	762 (82.6)	
Fertilization method, *n* (%)					0.537
IVF	575 (63.5)	565 (63.2)	618 (65.9)	594 (64.4)	
ICSI	206 (22.8)	223 (24.9)	199 (21.2)	217 (23.5)	
IVF + ICSI	124 (13.7)	106 (11.9)	121 (12.9)	111 (12.0)	
Duration of estrogen use (days)	12.7 ± 3.3	12.8 ± 3.2	12.8 ± 3.2	13.3 ± 3.5	0.001
Endometrial thickness (mm)	9.96 ± 1.97	9.93 ± 1.80	9.89 ± 1.92	9.66 ± 1.86	0.002
Progesterone level on embryo transfer day (ng/mL)	6.3 ± 1.4	8.8 ± 0.5	10.8 ± 0.7	15.3 ± 3.8	< 0.001
No. of embryos transferred, *n* (%)					0.165
Single	227 (25.1)	216 (24.2)	197 (21.0)	207 (22.5)	
Double	678 (74.9)	678 (75.8)	741 (79.0)	715 (77.5)	
Embryo stage at transfer, *n* (%)					0.010
Cleavage	798 (88.2)	769 (86.0)	842 (89.8)	784 (85.0)	
Blastocyst	107 (11.8)	125 (14.0)	96 (10.2)	138 (15.0)	
Transfer of ≥ 1 top-quality embryos (%)	191 (21.1)	157 (17.6)	170 (18.1)	168 (18.2)	0.209
Duration of embryo cryopreservation (years)	0.49 ± 0.50	0.46 ± 0.36	0.47 ± 0.42	0.52 ± 0.55	0.525

Data are presented as mean ± SD for all continuous variables. Abbreviations: BMI = body mass index; GnRH = gonadotropin-releasing hormone; IVF = in vitro fertilization; ICSI = intracytoplasmic sperm injection

**Table 2:** Pregnancy outcomes according to serum progesterone levels on the day of embryo transfer

	Progesterone quartiles (ng/mL)				*P*-value 1*	*P*-value 2*
	Q1 (< 7.9)	Q2 (7.9–9.6)	Q3 (9.7–12.0)	Q4 (≥ 12.1)		
Positive hCG test, n/N (%)	429/905 (47.4)	450/894 (50.3)	511/938 (54.5)	500/922 (54.2)	0.006	0.003
Implantation rate, n/N (%)	507/1583 (32.0)	526/1572 (33.5)	567/1679 (33.8)	545/1637 (33.3)	0.735	0.276
Clinical pregnancy rate, n/N (%)	393/905 (43.4)	414/894 (46.3)	451/938 (48.1)	445/922 (48.3)	0.136	0.030
Early miscarriage rate, n/N (%)	58/393 (14.8)	55/414 (13.3)	58/451 (12.9)	58/445 (13.0)	0.852	0.385
Ongoing pregnancy rate, *n/N* (%)	333/905 (36.8)	351/894 (39.3)	383/938 (40.8)	381/922 (41.3)	0.190	0.049
Live birth rate, *n/N* (%)	323/905 (35.7)	334/894 (37.4)	367/938 (39.1)	359/922 (38.9)	0.392	0.132

* *P*-value 1 for the comparison among Q1, Q2, Q3 and Q4. *P*-value 2 for the comparison between Q1 and Q2–Q4.

Abbreviations: hCG = human chorionic gonadotropin

In the multivariable analysis, the factors significantly associated with live birth rate were maternal age (adjusted OR = 0.91, 95% CI: 0.90–0.93, P < 0.001), maternal BMI (adjusted OR = 0.98, 95% CI: 0.95-1.00, P = 0.045), infertility diagnosis for males (adjusted OR = 1.40, 95% CI: 1.07–1.82, P = 0.014), duration of estrogen use (adjusted OR = 0.96, 95% CI: 0.94–0.99, P = 0.002), endometrial thickness (adjusted OR = 1.07, 95% CI: 1.02–1.11, P = 0.002), number of embryos transferred (adjusted OR = 2.01, 95% CI: 1.65–2.44, P < 0.001), and embryo stage at transfer (adjusted OR = 1.54, 95% CI: 1.21–1.97, P = 0.001) (Table 3).

**Table 3:** Relationship of risk factors with live birth by logistic regression analysis

	Adjusted OR (95% CI)	Wals	*P*-value
Maternal age (years)	0.91 (0.90–0.93)	111.46	< 0.001
Maternal BMI (kg/m^2^)	0.98 (0.95–1.00)	4.01	0.045
Duration of infertility (years)	0.99 (0.96–1.02)	0.4	0.526
Type of infertility			
Primary	Reference		
Secondary	1.06 (0.91–1.24)	0.59	0.442
Infertility diagnosis			
Tubal	Reference		
Male	1.40 (1.07–1.82)	6.05	0.014
Ovulatory	1.09 (0.89–1.34)	0.73	0.392
Endometriosis	1.05 (0.79–1.40)	0.12	0.735
Unexplained	1.09 (0.86–1.38)	0.52	0.469
Combined	0.97 (0.71–1.34)	0.03	0.861
Ovarian stimulation protocol			
Mild stimulation	Reference		
GnRH-agonist short	1.34 (0.91–1.96)	2.17	0.141
Progestin-primed ovarian stimulation	1.40 (0.99–1.98)	3.54	0.06
Fertilization method			
IVF	Reference		
ICSI	0.91 (0.75–1.10)	1.02	0.312
IVF + ICSI	1.16 (0.93–1.44)	1.70	0.192
Duration of estrogen use (days)	0.96(0.94–0.99)	9.7	0.002
Endometrial thickness (mm)	1.07 (1.02–1.11)	9.77	0.002
Progesterone level on embryo transfer day (ng/mL)			
Q1 (< 7.9)	0.89 (0.75–1.04)	2.09	0.148
Q2–Q4 (≥ 7.9)	Reference		
No. of embryos transferred			
Single	Reference		
Double	2.01 (1.65–2.44)	49.13	< 0.001
Embryo stage at transfer			
Cleavage	Reference		
Blastocyst	1.54 (1.21–1.97)	11.86	0.001
Transfer of ≥ 1 top-quality embryos (%)	1.09(0.89–1.33)	0.68	0.411
Duration of embryo cryopreservation (years)	0.89 (0.75–1.06)	1.67	0.197

Abbreviations: OR = odds ration; CI = confidence interval; BMI = body mass index; GnRH = gonadotropin-releasing hormone; IVF = in vitro fertilization; ICSI = intracytoplasmic sperm injection

Table 4 demonstrates the neonatal outcomes grouped by the serum P level on the day of ET. We did not find statistically significant differences in the neonatal outcomes, such as PTB, LBW, major congenital malformations and early neonatal death, across the serum P quartiles for either singletons or twins.

**Table 4:** Neonatal outcomes of live born infants according to serum progesterone levels on the day of embryo transfer

	Progesterone quartiles (ng/mL)				*P*-value
	Q1 (< 7.9)	Q2 (7.9–9.6)	Q3 (9.7–12.0)	Q4 (≥ 12.1)	
**Singletons**					
No. of children	232	247	283	278	
Gestational age (weeks)	38.6 ± 1.9	38.6 ± 1.5	38.7 ± 1.4	38.5 ± 1.8	0.739
Birthweight (g)	3362.0 ± 537.5	3378.5 ± 478.9	3406.1 ± 482.3	3351.6 ± 538.5	0.770
Preterm birth (< 37 weeks), *n* (%)	17 (7.3)	14 (5.7)	15 (5.3)	22 (7.9)	0.549
Low birthweight (< 2500 g), *n* (%)	13 (5.6)	9 (3.6)	8 (2.8)	16 (5.8)	0.262
Major congenital malformations, *n* (%)	2 (0.9)	2 (0.8)	5 (1.8)	5 (1.8)	0.664
Early neonatal death, *n* (%)	1 (0.4)	0 (0)	0 (0)	0 (0)	0.223
**Twins**					
No. of children	182	174	168	162	
Gestational age (weeks)	35.8 ± 2.4	35.6 ± 1.8	35.5 ± 2.6	35.6 ± 2.4	0.377
Birthweight (g)	2483.3 ± 462.5	2475.0 ± 407.5	2442.4 ± 526.4	2502.6 ± 432.5	0.801
Preterm birth (< 37 weeks), *n* (%)	92 (50.5)	102 (58.6)	92 (54.8)	78 (48.1)	0.223
Low birthweight (< 2500 g), *n* (%)	69 (37.9)	78 (44.8)	74 (44.0)	65 (40.1)	0.510
Major congenital malformations, *n* (%)	7 (3.8)	3 (1.7)	4 (2.4)	3 (1.9)	0.618
Early neonatal death, *n* (%)	2 (1.1)	0 (0)	1 (0.6)	2 (1.2)	0.576

Data are presented as mean ± SD for all continuous variables

## Discussion

The results of the present study showed that there was no association between serum P levels on the day of ET and the live birth rate in artificial cycles when using a combination of oral dydrogesterone and vaginal progesterone for LPS. After adjusting for all potential confounders, the relationship between serum P on the day of ET and the likelihood of a live birth was not present.

The importance of luteal P for the establishment and maintenance of pregnancy is undebatable, and it is well accepted that the success of frozen embryo transfer is crucially dependent on sufficient luteal phase support [35]. However, the optimal luteal P level following frozen embryo transfer is poorly understood and has not been consistent until now. There have been conflicting results when using intramuscular P. A recent study by Brady et al. indicated that P values lower than 20 ng/ml on the day of embryo transfer are associated with lower live birth and clinical pregnancy rates in donor recipient cycles [19]. Conversely, Kofinas et al. reported that P levels > 20 ng/ml on the day of transfer (during frozen single euploid embryo transfer cycles) were associated with a decreased OPR/LBR [23]. When using intravaginal P, different cut-off levels have been suggested. In a previous study [20], the optimal progesterone range needed to achieve a live birth was found to be 22-31ng/ml, while values outside this range were significantly related to lower pregnancy rates. Furthermore, two very recent studies found that there is an optimal serum P value around the time of ET (≥ 10 ng/ml) and on the day of the pregnancy test (≥ 11ng/ml) [21, 24]. In line with the above two studies, one study [22] showed that patients with serum P < 9.2 ng/ml on the day of ET after an artificial endometrial preparation cycle with vaginal micronized P had a significantly reduced OPR, which was observed in the oocyte donation cycle context. In the abovementioned studies using intravaginal P, although there are variations in the optimal serum P level, these results suggest that a minimal threshold of serum P on the day of ET needs to be reached.

Our endometrial preparation protocol is quite different in that it combines oral dydrogesterone and vaginal progesterone. Although 40 mg of dydrogesterone daily seems to be an enough dose for covering the needing of LPS in artificial cycles, it is not properly defined so far. That is why we are still combining the vaginal route. It is widely used in most IVF units in China [36, 37]. Therefore, we are interested in determining whether there is also an optimal serum P level when using a combination of oral dydrogesterone and vaginal progesterone. Consequently, the present study was designed, and we observed that there was no association between serum P levels on the day of ET and the live birth rate in artificial cycles when using a combination of oral dydrogesterone and vaginal progesterone for LPS.

In this study, we used dydrogesterone, which is an orally administered synthetic progestogen that has been successfully used for luteal phase support in stimulated IVF cycles over the past decade [16, 18, 38–40]. However, limited data are available about its use in artificial frozen–thawed cycles, which have different underlying endocrinological issues [41, 42]. Due to its unique molecular structure, dydrogesterone has a more selective binding capacity to the natural progesterone receptor. Therefore, much lower doses are required for dydrogesterone than for micronized progesterone [43]. Furthermore, because of the structural differences with progesterone, it cannot be quantified by any commonly used diagnostic test for measuring progesterone levels [16]. Consequently, the serum progesterone levels in this study only reflected vaginal progesterone administration. The P levels widely varied in this study, which were also observed in previous studies [20–22, 24]. Although all women receive the same dose of vaginal progesterone, the uptake, absorption and metabolism of each hormone can vary among women [20]. One study [41] reported comparable pregnancy rates between the oral dydrogesterone and micronized vaginal progesterone groups, using equivalent doses of 40 mg and 800 mg, respectively. In line with Rashidi’s research, our IVF unit administered 40 mg dydrogesterone orally. Therefore, the orally administered dydrogesterone probably covers the minimal threshold of serum P. Consequently, it may explain the results of the present study in which there was no association between serum P levels on the day of ET and the live birth rate in artificial cycles using a combination of oral dydrogesterone and vaginal progesterone for LPS. To our knowledge, this is the first study to estimate the association between serum P levels on the day of ET and the live birth rate using a combination of oral dydrogesterone and vaginal progesterone for LPS. The oral route of administration is thought to be a more patient-friendly regimen that lacks the side effects of vaginal or intramuscular administration. However, whether only 40 mg dydrogesterone is adequate in AC-FET for LPS remains to be determined. Although two small clinical studies have investigated the use of 30 or 40 mg oral dydrogesterone for luteal phase support in programmed frozen-thawed cycles [42, 44], there is also lack of randomized controlled trials at least at the national level for determining the required dose for oral dydrogesterone. Our luteal phase support protocol reduces the vaginal dose to a level that is more comfortable for patients, also ensures progesterone support for patients with poor vaginal absorption. There is much literature showing the relevance of measuring serum P in artificial cycles when using vaginal progesterone. But in this study it cannot be appreciated that if there is any impact of the levels of serum P according to the vaginal administration because all patients were already receiving dydrogesterone and thus, “protected” to the possible situation of bad absorption of vaginal progesterone.

Previous studies have reported that a high BMI, advanced maternal age or prolonged estrogen treatment are associated with a lower live birth rate, which in line with our study [45–47]. The main limitation of our study is the retrospective nature of the data. Although we adjusted our analysis to minimize the likelihood of confounding, selection bias could not be completely ruled out. Additionally, embryo aneuploidy is one of the main aspects related to implantation failure, so we have not ruled out the impact of embryo quality on this finding. Moreover, there may be a serious confounding bias in the results of this study in which there was no association between serum P levels on the day of ET and the live birth rate in artificial cycles using a combination of oral dydrogesterone(40 mg/d) and vaginal progesterone(400 mg/d) for LPS because patients were supported with combination of oral dydrogesterone and vaginal progesterones, but serum progesterone levels responded only to the latter. In other words, the orally administered 40 mg dydrogesterone probably covers the minimal threshold of serum P. So different findings may be observed if the amount of oral dydrogesterone is reduced.

## Conclusions

This is the first study to estimate the association between serum P levels on the day of ET and live birth in artificial cycles using a combination of oral dydrogesterone(40 mg/d) and vaginal progesterone(400 mg/d) for LPS. We found that the serum P levels on the day of ET do not correlate with live birth using the combination progesterone for LPS. In addition, prospective, randomized, controlled, blinded trials are merited to determine the optimal dosing regimen for oral dydrogesterone in in AC-FET for LPS.

## Electronic supplementary material

Below is the link to the electronic supplementary material.

Additional file 1: Supplementary Figure 1

## Data Availability

The data used and analyzed during the study are available from the corresponding author if the request is reasonable.

## List of Abbreviations

P: Progesterone
FET: Frozen-thawed embryo transfer
ET: Embryo transfer
LB: Live birth
AC: Artificial cycles
LPS: Luteal phase support
E2: Estradiol
OR: Odds ratio
CI: Confidence interval
ART: Assisted reproductive technology
IVF: In vitro fertilization
ICSI: Intracytoplasmic sperm injection
MVP: Micronized vaginal progesterone
hCG: Human chorionic gonadotrophin
CPR: Clinical pregnancy rate
OPR: Ongoing pregnancy rate
PTB: Preterm birth
LBW: Low birthweight
PPOS: Progestin-primed ovarian stimulation
