# Supplementary material for: Association between serum progesterone levels on the day of frozen-thawed embryo transfer and pregnancy outcomes after artificial endometrial preparation
Source: BMC Pregnancy Childbirth. 2023 May 30;23:401. doi: 10.1186/s12884-023-05596-4 (PMC10227978; doi:10.1186/s12884-023-05596-4)
Supplement: Supplementary file 1 — Additional file 1: Supplementary Figure 1 [file 12884_2023_5596_MOESM1_ESM.docx]

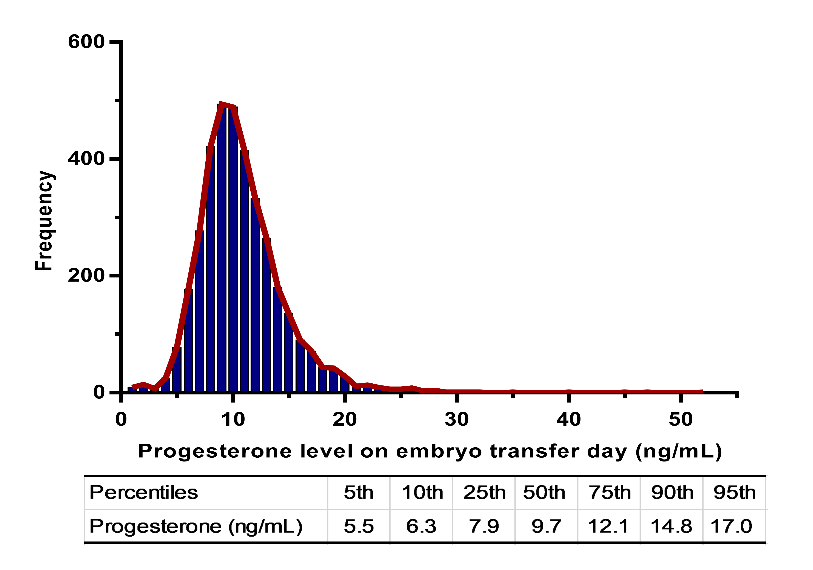


**Supplementary Figure 1.** Frequency distribution of serum progesterone level on the day of embryo transfer day in frozen–thawed embryo transfer cycles.
